# Machine-learned-based prediction of lower extremity overuse injuries using pressure plates

**DOI:** 10.3389/fbioe.2022.987118

**Published:** 2022-09-02

**Authors:** Loren Nuyts, Arne De Brabandere, Sam Van Rossom, Jesse Davis, Benedicte Vanwanseele

**Affiliations:** ^1^ DTAI, Department of Computer Science, KU Leuven, Leuven, Belgium; ^2^ Human Movements Biomechanics Research Group, Department of Movement Sciences, KU Leuven, Leuven, Belgium

**Keywords:** running, machine learning, prediction, lower extremity overuse injuries, pressure plate, plantar pressure

## Abstract

Although running has many benefits for both the physical and mental health, it also involves the risk of injuries which results in negative physical, psychological and economical consequences. Those injuries are often linked to specific running biomechanical parameters such as the pressure pattern of the foot while running, and they could potentially be indicative for future injuries. Previous studies focus solely on some specific type of running injury and are often only applicable to a gender or running-experience specific population. The purpose of this study is, for both male and female, first-year students, (i) to predict the development of a lower extremity overuse injury in the next 6 months based on foot pressure measurements from a pressure plate and (ii) to identify the predictive loading features. For the first objective, we developed a machine learning pipeline that analyzes foot pressure measurements and predicts whether a lower extremity overuse injury is likely to occur with an AUC of 0.639 and a Brier score of 0.201. For the second objective, we found that the higher pressures exerted on the forefoot are the most predictive for lower extremity overuse injuries and that foot areas from both the lateral and the medial side are needed. Furthermore, there are two kinds of predictive features: the angle of the FFT coefficients and the coefficients of the autoregressive *AR* process. However, these features are not interpretable in terms of the running biomechanics, limiting its practical use for injury prevention.

## 1 Introduction

With the growing awareness of physical activity for a healthy lifestyle, running has become increasingly popular. It is beneficial for both the physical and mental health (Penedo and Dahn (2005); Warburton et al. (2006); Warburton and Bredin (2017); Koplan et al. (1982); Major (2001)). However, like all physical activity, it comes with an associated risk of becoming injured. In turn, injuries results in negative physical, psychological and economical consequences (Melzer et al. (2004); Walker et al. (2007); van Mechelen (1992); Hespanhol Junior et al. (2017, 2016); Koplan et al. (1982); Major (2001)), which emphasizes the importance of injury prevention (Eetvelde et al. (2021); Emery and Pasanen (2019); Beato et al. (2021); Hespanhol Junior et al. (2017); Cloosterman et al. (2020)).

Overuse injuries are one of the most common types of injury, and they may account for up to 80% of running-related injuries (Lopes et al. (2012)). Injuries can arise due to several factors and their complex interaction. These factors can be person-specific such as age, gender, weight, injury history, fitness level and gait of the person (Adirim and Cheng (2003); Rolf (1995); Bahr and Krosshaug (2005); Olivier et al. (2015)). These factors can also result from choices such as training errors or the use of inappropriate equipment (Adirim and Cheng (2003); Rolf (1995)). Some of them can be adapted such as the gait, training and equipment and therefore have a large potential for injury prevention and prediction (Adirim and Cheng (2003); Hreljac (2004)). Especially the effect of the running gait on the development of running related overuse injuries has been extensively investigated (Chan et al. (2018); Napier et al. (2015); Dugan and Bhat (2005)). A recent systematic review (Ceyssens et al. (2019)) identified sixteen studies that investigated biomechanical risk factors and their association to running-related injuries (RRIs). However, results from these studies are inconclusive and provide only limited evidence for a few biomechanical factors as risk factor for some specific injuries and even in a specific population (gender specific or running experience specific). One of the main challenges of biomechanical studies to identify risk factors for overuse injuries is to be able to measure large groups as 3D motion analysis is quite time consuming and requires expensive, often lab-based, equipment. Plantar pressure plates can be used in the field in a quick and easy way to obtain data on the pressure distribution underneath the feet, the landing pattern, vertical ground reaction force and the foot roll-off. Therefore, plantar pressure measurement opens the potential to measure large groups and use more complex data analysis techniques, which might help to predict overuse injuries.

Machine learned models have recently attracted more attention in injury prediction because of their high predictive performance. Starting from a training dataset, a machine learned model learns the relationship between the input features and the target variable. When the model is deployed, it uses its knowledge about the relationship to predict the target variable for new instances. In recent years, several machine-learned injury-predicting models (Wilzman et al. (2022); Booth et al. (2020); Bogaert et al. (2022); Eetvelde et al. (2021); Rossi et al. (2018); Ayala et al. (2019); Lövdal et al. (2021); Martínez-Gramage et al. (2020); Carey et al. (2018); Rommers et al. (2020); Oliver et al. (2020b)) and machine-learned models based on plantar pressure (Wilzman et al. (2022); Booth et al. (2020); Chen et al. (2021); Ardhianto et al. (2022); Botros et al. (2016); Nong et al. (2021); Jeon et al. (2008)) have been proposed and motivated the use of machine learning in this study. However, most of the proposed injury-predicting machine learning models focus on elite athletes in one particular sport such as soccer (Rossi et al. (2018); Ayala et al. (2019)), running (Lövdal et al. (2021); Martínez-Gramage et al. (2020)) or football (Carey et al. (2018); Rommers et al. (2020); Oliver et al. (2020b)) and the insights gained in these studies might not be transferable to other sports. Moreover, Winter et al. (2019) showed that factors that play a role in injury development depend on the skill level of the participants, which indicates that the findings from the aforementioned studies might not apply to non-elite athletes. Likewise, Videbæk et al. (2015) reported that the incidence of RRIs per 1000 h of running differs significantly for novice and recreational runners, with a value of 17.8 (95 % CI 16.7–19.1) for novice runners and 7.7 (95 % CI 6.9–8.7) for recreational runners. In addition, Ceyssens et al. (2019) concluded from their systematic review that gender should be taken into account to study biomechanical risk factors associated with running-related injuries. As several studies focus solely on male athletes (Oliver et al. (2020b); Rossi et al. (2018); Rommers et al. (2020); Franklyn-Miller et al. (2013)), results are not directly transferable to female athletes. Other studies (Wilzman et al. (2022); Bogaert et al. (2022)) focus on both male and female runners, but the machine-learned models are trained separately for each gender. This increases the amount of data that is needed since two separate models have to be trained and common risk factors for both men and women have to be learned separately in each model. This increases the need for one machine learning model that can predict injuries for both men and women.

Therefore, the aim of this study was to develop a machine learning model that is able to predict the risk of lower extremity overuse injury development in both males and females based on a baseline running assessment using plantar pressure data. A lower extremity overuse injury is defined as an injury that is caused by a high physical load because of an incomplete repair process and with a gradual onset. It is characterized by progressive symptoms, the absence of a known single traumatic event and the lack of a recovery period (Nesterovica (2020)). Subjects were prospectively followed during a 6 month period with similar loading, to answer the next two key questions for a study population consisting of male and female, first-year students:

1) *How accurately can the development of a lower extremity overuse injury be predicted based on foot pressure measurements?*
2) *Which loading features are predictive for lower extremity overuse injury development?*

## 2 Data

### 2.1 Participants

In total, 249 first-year bachelor students from two separate year cohorts (2019–2020 and 2020–2021) from the movement sciences program at KU Leuven in Belgium participated in this study. However, only participants that suffered a lower extremity overuse injury (35 subjects) or that did not get injured (120 subjects) were included. Participants that suffered an acute injury (35 subjects), had an unknown injury status (6 subjects) or had missing/incorrect values (53 subjects) were excluded for further analysis. Table 1 reports the gender, length and weight for the 155 included participants. All of them are around 18–19 years old. Table 2 provides a more detailed overview of the different injuries of the included participants.

**TABLE 1:** Statistics about the available dataset. The average is denoted as *μ* and the standard deviation as *SD*. Only the healthy people and the ones with lower extremity overuse injuries are included. All people that had an unknown injury type, missing/incorrect values or an acute injury are omitted from this table.

	Gender		Length [cm]		Weight [kg]		Total
	*Male*	*Female*	*μ*	*SD*	*Μ*	*SD*	
Healthy	83	37	175.69	8.23	68.65	9.00	120
Injured	26	9	177.70	8.04	69.75	8.63	35

**TABLE 2:** Number of people suffering from a specific lower extremity overuse injury. Note that some people had multiple injuries, so the total number of injuries does not equal the total number of injured people. All people that had an unknown injury type, missing/incorrect values or an acute injury are omitted from this table.

	Number of people with injury
Medial tibial stress syndrome (MTSS)	21
Pain complaints with regard to groin, knees and ankles	1
Plantar fasciopathy	1
Patellofemoral suffering	3
Tendinopathy	6
Musculo-ligamentary overload complaints	1
Strain on hamstrings	1
Pain in hip socket	1
Iliotibial band syndrome (ITBS)	1
Adductors	1
Soft tissue overload ankle	1

All students participated on a voluntary basis, without any positive or negative consequences associated with their engagement. The study was conducted according to the guideline of the Declaration of Helsinki and approved by the Ethics Committee of UZ Leuven in Belgium (protocol code: S60810, date of approval: 25 October 2017). All subjects involved gave their informed consent. All students followed the same academic sports program at a common sport facility for 26 weeks per academic year. Sports included several team sports such as soccer, handball, basketball, volleyball, and individual sports such as track and field, gymnastics, dance and swimming. The weekly program consisted of 10 hours of sports on average. Students were required to report all injuries to the sport medicine physician of the Sport Medical Advise Center (University Hospital Leuven).

After 6 months, the physicians communicated for each participant whether an injury had occurred. This is the case when either a reduction in the amount of physical activity is recommended or medical advice or treatment was needed (Aristizábal Pla et al. (2021)). Furthermore, it was established whether the injury was a lower extremity overuse injury. Nesterovica (2020) defines a lower extremity overuse injury as an injury that is caused by a high physical load because of an incomplete repair process and with a gradual onset. It is characterized by progressive symptoms, the absence of a known single traumatic event and the lack of a recovery period. Injuries that were not consistent with the above definition of a lower extremity overuse injury were classified as acute injuries and not included in this study.

### 2.2 Data collection

At the start of the academic year, all participants had to run over a pressure plate (Materialise Motion 1x0.4m, sampling at 250 Hz). First, five strides from each foot were collected by instructing the participants to walk over the pressure plate using their normal walking gait. Second, five strides from each foot were collected for each subject by running barefoot over the plate at their own pace. Finally, five strides from each foot were collected for each subject by running over the plate with their own running shoes on. In addition to that, each participant filled in a questionnaire including questions about their weight, length, shoe size, previous injuries, dominant leg, whether they have insoles, etc. The BMI is derived from their weight and height and also added to it.

### 2.3 Pressure and force measurements

The data were analyzed using the scientific version of the footscan Suite (footscan V9, Materialise Motion, Belgium). For each trial, the software automatically divides the foot in ten anatomical zones. These areas were defined as the hallux (toe 1), toes 2-5, metatarsal heads 1 to 5 (separate zones), midfoot, medial heel and lateral heel. From the pressure plate, we extracted four main outcome parameters for each point in time:

• **Vertical force** The vertical force is the net vertical force acting on the foot.
• **Peak pressure** This is the maximal pressure in a foot area at each time point.
• **Mean pressure** This is the average over all pressure values within a foot area at each time point.
• **Mean force** This is derived by multiplying the mean pressure with the area of its corresponding foot area.

For the first, the data was considered for the whole foot, whereas for the last three, the parameters were determined for each of the ten anatomical zones. Only the running data was used as input for the machine learning pipeline, whereas the walking data was used to rescale the running data (Section 3.1.2).

## 3 Machine learning pipeline

Our goal is to predict the probability that a subject will develop a lower extremity overuse injury in the following 6 months based on the pressure, force and vertical force measured during barefoot and shod running. To this end, we train a logistic regression model as it is a simple, well-known model (Cramer (2002); Bender and Grouven (1997)). Because of the limited amount of data, neural networks are not an option, but logistic regression models can still produce accurate results in that case. Furthermore, previous studies have successfully applied logistic regression to planter pressure data (Forghany et al. (2019); Ménard et al. (2021)). It is also straightforward to determine the importance of each feature in a logistic regression model, which makes it suitable for finding the predictive loading features (second research question).

**FIGURE 1:**
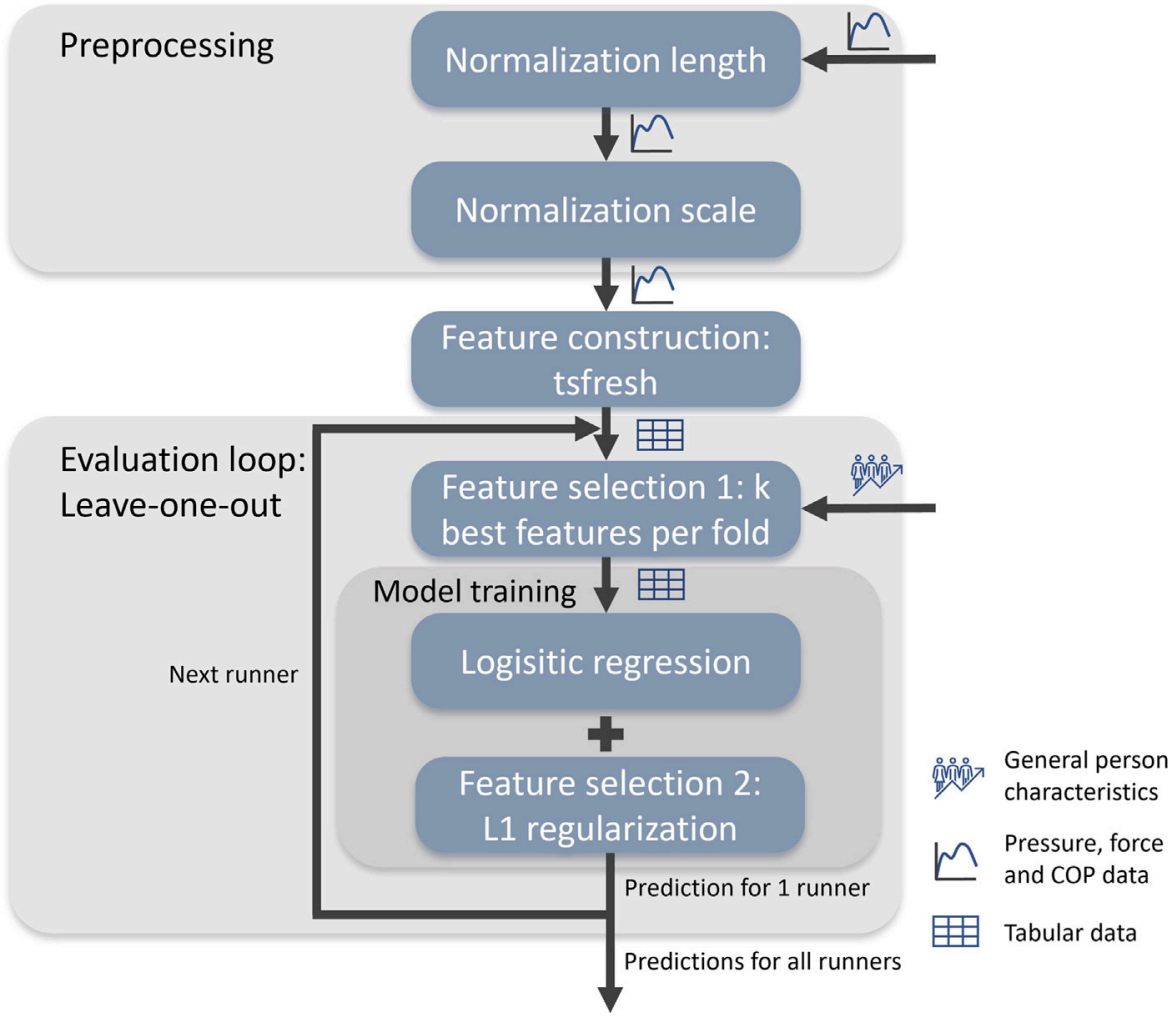
The machine learning pipeline used to predict lower extremity overuse injuries, including the evaluation process. Evaluation is done with leave-one-out cross validation. The final results are averaged over the results of each single prediction.

To train a model, we employ the pipeline illustrated in Figure 1. This pipeline has the following key steps, which will be described in more detail in the following sections:

1) The *preprocessing* step optimally aligns all trials by making them equally long and rescaling them (Section 3.1).
2) The *feature construction* step extracts features from the time series data to transform the data into the tabular format expected by classic machine learning methods (Section 3.2).
3) The *feature selection* step determines the most important features (Section 3.3). First the best number of features *k*_*best*_ per fold are determined. Based on this, the *k*_*best*_ most predictive features per fold are selected. Finally, L1 regularization is performed during the training of the logistic regression model in the model training step as an additional form of feature selection.
4) In the *model training* step, a logistic regression model is trained on the *k*_*best*_ most predictive features (Section 3.2). The model training step additionally performs a second form of feature selection by using L1 regularization.
5) The *evaluation* of the machine learning pipeline is done with a leave-one-subject-out approach (Section 3.2). This work reports the area under the receiver-operator characteristic (ROC) curve and the Brier score.

### 3.1 Preprocessing

There are two challenges to contend with in the raw data. First, each subject completed multiple trials and we need to aggregate the data into a single set of signals to analyze. The most natural way to do this is by averaging the trials. This is complicated by the fact that each trial is of a different length. Consequently, different stages during running (first contact with ground, push-off, etc.) can differ between different trials. Averaging over the raw data would mix the different stages which results in a less stable average with even more noise than the original data. Therefore, we first align the data from each trial and make them equal length (Section 3.1.1).

The second challenge aims to avoid noise coming from different body weight and running speeds. It is solved by rescaling all measurements while still keeping the relative scale difference between different foot areas (Section 3.1.2) so that no valuable information is lost.

#### 3.1.1 Normalisation length of measurements

To make all trials of all subjects equally long and optimally aligned, the following steps were performed.

1) We start by looking for the longest trial amongst all subjects for each kind of footwear (barefoot, shod). These two trials become the references for their corresponding footwear condition and are shared for all subjects and measurement types (mean pressure/force, peak pressure).
2) The following steps are performed for each trial separately:
  a) We use padding to make the trial the same length as the footwear-specific reference. All possible ways of adding zeros to the beginning, end or some combination of both are tried.
  b) After the previous step, there are multiple padded trials to replace the unpadded one. We choose the one that maximizes the 2D histogram-based mutual information (256 bins in each dimension)^1^ (Booth et al. (2020)) between the padded signal and its corresponding reference. This way, the padded signal is optimally aligned with respect to the reference trial, while keeping all time-related elements of the trial unchanged. This is not the case if the unpadded signal would have been stretched or compressed to match a certain length.

**FIGURE 2:**
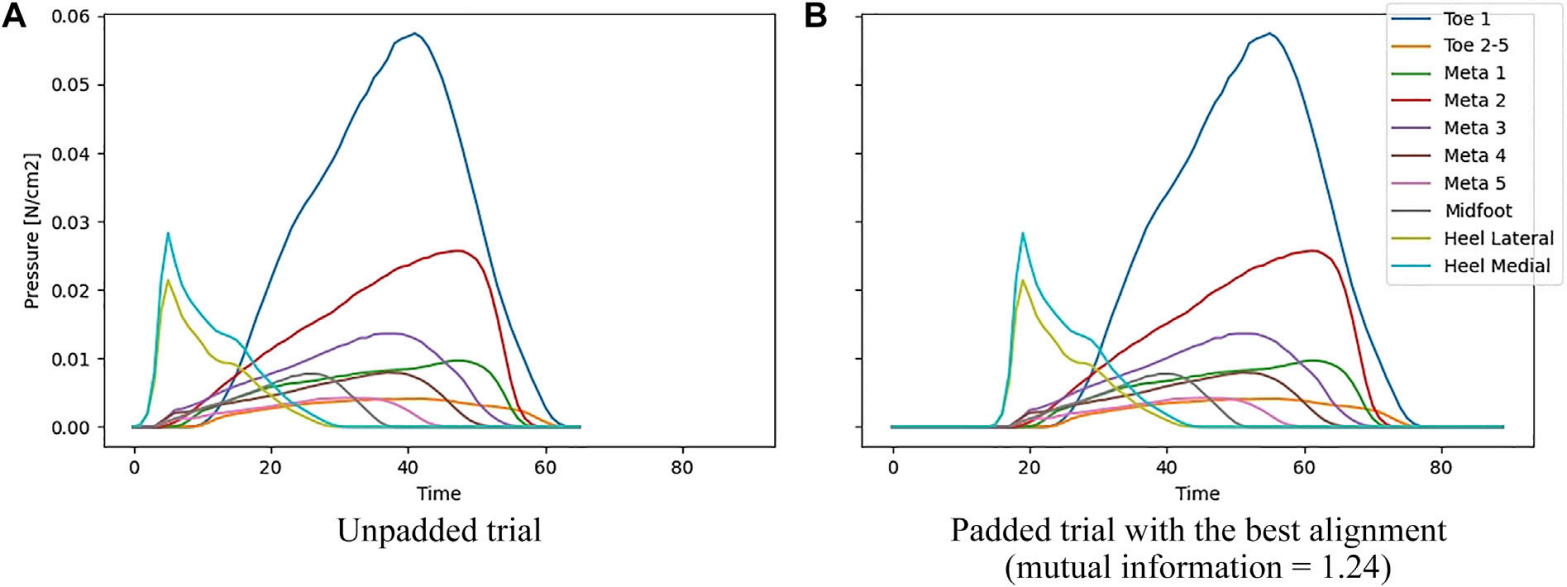
a) Unpadded trial. b)Padded trial with the best alignment (mutual information = 1.24). **(A)** shows an unpadded trial, **(B)** shows the padded trial that is optimally aligned (it has the highest 2D histogram-based mutual information) w.r.t. its reference.

Figure 2A shows an example of an unpadded trial and Figure 2B shows the corresponding padded trial which has the optimal alignment with respect to the reference.

#### 3.1.2 Normalisation scale of measurements

To normalize the differences in running speed and the participant’s weight, the *peak pressure*, *mean pressure* and *mean force* measurements are scaled with respect to the maximum vertical force while walking barefoot of the corresponding participant. For each person, all these measurements are divided by his/her maximal vertical force value, which can easily be retrieved from the *vertical force* measurements. This results in a dataset where each type of measurement has more or less the same scale.

### 3.2 Feature construction and model training

#### 3.2.1 Feature construction

Standard machine learning algorithms such as logistic regression are not applicable to raw time series data as they only operate on features describing these time series. Therefore, we use the Python library *tsfresh* (Christ et al. (2018)) to extract those features from the time series and put them in a tabular format. We used the default setting which extracts features^2^ such as the maximum, absolute energy, linear trends, autocorrelation, FFT coefficients, etc. We extracted these features from all outcome parameters (peak pressure, mean force/pressure and vertical force) from each of the ten foot regions if applicable. In total, this yields 22,180 features for each subject.

#### 3.2.2 Model training

We learn the model using the LIBLINEAR solver (Fan et al. (2008)) with maximum 100 iterations using L1 regularization with the default regularization strength of the *LogisticRegression* class of scikit-learn (scikit-learn, RRID:SCR_002,577) version 1.0.2. The L1 regularization can be seen as a second type of feature selection and will be further explained in Section 3.3.2.

### 3.3 Feature selection

Machine learned algorithms struggle with large numbers of features. Therefore, we employed two types of feature selection to reduce the number of considered features: the first one selects the best number of features *k*_*best*_ per fold and afterwards the *k*_*best*_ most predictive features per fold. This is done separately for each fold to avoid data leakage. The second type of feature selection is done by applying L1 regularization in the model training phase.

#### 3.3.1 k most predictive features per fold

First, we employ a filter-based feature selection separately on each fold. We use *SelectKBest* class of scikit-learn (scikit-learn, RRID:SCR_002,577) version 1.0 which produces a rank-ordered list of the most informative features using a statistical test. We vary *k* ∈ {5, 10, 15, 20, 25} and train a logistic regression including the *k* highest scoring features. We then pick the value of *k* that results in the highest train set AUC score (Area Under the receiver-operator Characteristic (ROC) curve) for that fold. This resulted in an average of 23 features per fold and a median of 25. It is essential to note that this is repeated separately for each fold of the cross-validation procedure to avoid leakage of information between the train and test set. Failing to do this would result in overoptimistic estimates of performance.

We considered 5 and 25 features because it balances the risk of overfitting by using too many features and throwing away too much valuable information by using too few features. A logistic regression model has a high chance of overfitting when the number of features is larger than 10% of the number of samples (the 10%-rule) (Concato et al. (1995); Peduzzi et al. (1995, 1996)), although this rule can be relaxed a bit (Vittinghoff and McCulloch (2006)). Additionally, the steps of 5 features is precise enough to adapt the number of features to each fold, while also not being computationally too demanding.

#### 3.3.2 L1 regularization

During the model training step, L1 regularization is applied to the logistic regression model. Because L1 regularization will force the coefficients associated with less predictive features to be zero, it can be seen as a second type of feature selection that is applied during the model training step. In our current implementation, there were on average 5 features that had non-zero coefficients after training the model with the L1 regularization.

### 3.4 Evaluation

The evaluation is divided in two parts. The first part concerns the evaluation of the pipeline and the predictions made by it and answers the first key question of this paper. The second part looks into the importance of the different foot areas, measurement types (mean pressure/force, peak pressure, vertical force) and footwear (barefoot, shod) and partially answers the second key question.

#### 3.4.1 Evaluation of pipeline

Because the data contains a small number of subjects, we perform leave-one-out cross validation. This means that for each runner, a model is trained using the entire dataset except that one runner. The data for the held-aside runner then serves as the single test example that the learned model makes a prediction for. We consider two evaluation metrics. First, we look at the area under the receiver-operator characteristic (ROC) curve (Bradley (1997); Marzban (2004)) or AUC. This metric evaluates a model’s ability to rank examples. Second, we report the Brier score (Brier (1950); Rufibach (2010)) which is computed as:

$$BS=\frac{1}{N}\overset{N}{\underset{i=1}{\sum }}{\left(p_{i}-y_{i}\right)}^{2}$$ (1)

where *N* is the number of samples, *p*_*i*_ is the predicted probability of sample *i* to be injured and *y*_*i*_ is the label of sample *i* (0 for healthy and 1 for injured). This metric evaluates how well calibrated the learned model’s probabilities are, with a Brier score of 0 for perfectly calibrated probabilities. A probability is calibrated if it reflects the true likelihood of events. For example, if the model predicts that 10 participants are all healthy with a probability of 80%, then we expect that 8 participants are healthy and two are injured.

#### 3.4.2 Importance foot areas, measurement types and footwear

For the first part of the second key question of this paper, we investigated which foot areas, measurement types (mean pressure/force, peak pressure, vertical force) and footwear (bare, shod) are important for the predictions. Each one of these forms a “group” of features that contains all features that are based on it. For example, the group of features of metatarsal 1 contains all features that are derived from all measurements (mean pressure/force, peak pressure, vertical force, barefoot and shod) involving metatarsal 1. Some groups may overlap, like the *mean force* group and the *metatarsal 1* group, while others are disjunct, like the *metatarsal 1* group and the *metatarsal 2* group.

For each group of features, a logistic regression model (same settings as described in Section 3.2) is trained on 15 features, as selected by the *SelectKBest* class of scikit-learn, but where the features derived from the considered group are excluded. Training so many models on the *k*_*best*_ most predictive features per fold as described in Section 3.3.1 would be computationally infeasible, so instead we applied the 10%-rule (Concato et al. (1995); Peduzzi et al. (1995, 1996)) and trained these models on 0.1*155 ≈ 15 features.

Finally, the model that could choose the 15 most predictive features between all features can be compared to each model where one group of features was excluded to determine the impact of that group of features on the predictions.

## 4 Results

The machine learning pipeline given in Figure 1 can predict lower extremity overuse injuries with an AUC of 0.639 and a Brier score of 0.201. The AUC score implies that the model can distinguish reasonably well between healthy and injured runners, while the low Brier score implies that the model is well calibrated.

Table 3 compares the models where each time one group of features was excluded and reports the changes in AUC and Brier score, where a positive change implies improvement of the model when that group of features is omitted. The table shows that:

• Toes 2-5 and metatarsal 1 and 3 are the three most important foot areas.
• The peak pressure is more important than the mean pressure and the mean force.
• The general person characteristics, medial heel and vertical force are never chosen in the feature selection steps, which explains their difference of 0.

**TABLE 3:** The ranking and the improvements in the AUC and Brier scores for each foot area, measurement type and footwear when 15 features are selected by *SelectKBest*. A lower rank indicates a more important group of features. *Δ* refers to the improvement in the corresponding score with respect to the model that has access to all features, where a positive improvement means that the model where the current group of features was excluded performed better than the model that had access to all features.

	Rank	Excluded group of features	*Δ* AUC	*Δ* Brier
Foot area	1	toes 2–5	−1.81e-2	−2.2e-2
	2	metatarsal 1	−2.55e-2	−7.58e-3
	3	metatarsal 3	−4.05e-3	−9.91e-4
	4	medial heel	0	0
	5	lateral heel	9.52e-3	1.88e-3
	6	midfoot	1.14e-2	4.33e-3
	7	metatarsal 4	3.19e-2	4.86-e3
	8	toe 1	2.79e-2	1.51e-2
	9	metatarsal 2	5.17e-2	7.4e-3
	10	metatarsal 5	5.93e-2	3.46e-2
Measure-ment	1.5	general person characteristics	0	0
	1.5	vertical force	0	0
	3	peak pressure	2.14e-2	7.07-e3
	4	mean force	2.29e-2	2.05e-3
	5	mean pressure	2.67e-2	1.18e-2
Foot-wear	1	shod	6.21e-2	3.37e-2
	2	barefoot	1.05e-1	3.46e-2

The number of times each feature was chosen by the *SelectKBest* class and the L1 regularization was summed across the different folds and the features that were present in at least 10% of the folds are displayed in Table 4, together with the percentage of folds they occur in. The following definitions will further clarify some concepts used in Table 4 ^3^.

**TABLE 4:** Features that are present in at least 10% of the folds. The “occurrence” column gives the percentage of the folds where the feature was chosen by *SelectKBest* and the L1 regularization. Definition 1 and 2 further explain some used terminology.

Foot area	Measurement	Footwear	Feature	Occurrence (%)
toe 1	mean force	Shod	angle of FFT coefficient 86	43.2
toes 2–5	mean pressure	Shod	angle of FFT coefficient 21	100
	peak pressure	Shod	angle of FFT coefficient 21	95.5
metatarsal 2	peak pressure	Shod	7th coefficient of the autoregressive *AR* process, with maximum lag 10	95.5
metatarsal 3	peak pressure	Barefoot	7th coefficient of the autoregressive *AR* process, with maximum lag 10	35.5
metatarsal 5	peak pressure	Barefoot	angle of FFT coefficient 31	100

*Definition 1. The FFT coefficients of a time series *X* with length *n* is defined as*

$$A_{k}=\overset{n-1}{\underset{m=0}{\sum }}X_{m}\exp\left(-2\pi i\frac{mk}{n}\right),\;\;k=0,\ldots ,n-1$$ (2)

*Definition 2. The autoregressive process* *AR*(*k*) *with coefficients* *ϕ*_*i*_ (*i* = 0, … , *k*)*, maximum lag* *k* *and error* *ϵ* *of a time series* *X* *, is defined as*

$$X_{t}=\phi_{0}+\overset{k}{\underset{i=1}{\sum }}\phi_{i}X_{t-i}+\epsilon_{t}$$ (3)

Four features in Table 4 are chosen very consistently, they are present in almost every fold. The features in Table 4 itself are also very consistent: only the angle of FFT coefficients and the coefficients of the autoregressive process *AR* (*k* = 10) with maximum lag 10 are needed.

## 5 Discussion

This paper looks into two key questions which are covered in Section 5.1 and Section 5.2 respectively. For a study population consisting of male and female, first-year students:

1) How accurately can lower extremity overuse injuries be predicted based on foot pressure measurements?
2) Which loading features are predictive for lower extremity overuse injury development?

### 5.1 Performance of the model

The developed machine learning pipeline using a running assessment on a pressure plate is able to predict running related overuse injuries in a general physically active population with an AUC of 0.639 and a Brier score of 0.201. This demonstrates the potential of using pressure plate measurements in combination with a machine learning model to identify people at risk of lower extremity overuse injuries. The obtained results of our current model are comparable to the performance of models described in the literature of injury prediction models (Jauhiainen et al., 2021; Oliver et al., 2020a). Results of this model are also similar to a previous attempt in a similar population (Bogaert et al., 2022). In that study a trunk-based 3D accelerometer was used during an all-out running test. However, two separate models for men and women were trained, whereas we used a single model to predict injuries for both men and women.

### 5.2 Predictive loading features

#### 5.2.1 Predictive foot areas

Table 3 shows that the toe 2-5, metatarsal 1 and metatarsal 3 are the most predictive foot areas for lower extremity overuse injuries. It is important to note that the results of Table 3 do not take interdependencies between features into account. Having a positive improvement when the group of features is left out, does not necessarily mean that it is better to permanently omit these groups. Some groups can share some piece of information which causes the predictions to improve if one is left out because the model then learns from fewer and less correlated features. Removing all groups with that piece of information however might seriously deteriorate the predictions.

Contrary to Table 3, Table 4 shows that features based on metatarsal 3 are only present in 35.5% of the folds and thus don’t contribute that much to the predictions. Features based on metatarsal 1 are present in less than 10% of the folds. Features based on metatarsal 2 and 5 however are present in almost every fold. This might indicate that there is some redundant information in the features based on the metatarsals and that not all of them are needed to make accurate predictions. Willems et al. (2007) found that the people with overuse injuries exert more pressure on the medial side of the foot than on the lateral side. Our results confirm that both the lateral and medial side of the foot are important as in both tables, parts of the medial (toe 1, metatarsal 1 and 2) and parts of the lateral side (toes 2-5, metatarsal 5) are found to be the most predictive. However, we cannot conclude whether the injured participants exert more pressure on the lateral or medial side of the foot because the features that are used to train the model (see Table 4) are not directly interpretable in terms of higher or lower pressure.

Both Table 3 and 4 suggest that only information from the forefoot is needed to predict lower extremity overuse injuries. Neither the midfoot nor the heel are found to be important predictors. This is similar to the findings of Willems et al. (2007) as differences in pressure where detected at forefoot flat and at heel-off. This indicates that the push-off phase is more crucial for lower extremity overuse injuries than the first contact, which coincides with the moment of highest ground reaction forces imposed on the foot.

#### 5.2.2 Predictive measurements

Both Table 3 and 4 agree that the peak pressure is the most predictive measurement. The general person characteristics and vertical force are never chosen in the feature selection steps, so the only conclusion we can draw is that the *SelectKBest* class and the L1 regularization see them as the least predictive of all. The high predictive performance of the peak pressures indicates that the higher pressures are crucial for lower extremity overuse injury development.

#### 5.2.3 Predictive footwear

From Table 3 and 4 we can conclude that both barefoot and shod data are important predictors for lower extremity overuse injuries. Barefoot data contains more fine-grained pressure information and can thus tell us more about the exact gait of the person. Shod data on the other hand takes the shoes into account, which might (partially) correct an incorrect gait.

#### 5.2.4 Predictive features

Table 4 shows that only two kinds of features are used: the angle of a FFT coefficient and the 7th coefficient of the autoregressive *AR* process with maximum lag 10. This is quite surprising as *tsfresh* computes a lot of different features which are barely chosen in the feature selection steps. At the same time, this also shows the difficulty with the interpretation of these type of features. The angle of a FFT coefficient is very hard to associate with a specific running pattern. This therefore means that when using our model to identify runners at risk we will need to perform additional analysis to determine potential interventions or treatments. Furthermore, based on these features, we cannot make any conclusions about overload or underload of certain regions of the foot, which limits the interpretability of the model.

### 5.3 Strengths and limitations

Our proposed method has an AUC (0.639) score that is comparable to the performance of the models described in the literature of injury prediction with machine learning (Jauhiainen et al., 2021; Oliver et al., 2020a; Bogaert et al., 2022) In contrast to Jauhiainen et al. (2021) and Oliver et al. (2020a), only a small minority of the participants were elite athletes, which makes our approach more applicable to the wider public. Compared to Bogaert et al. (2022), we used a single model for the prediction of both male and female participants which needs less data to train the model and learns the common risk factors for both men and women.

However, our proposed method also has several limitations. First, the features that were found to be predictive are not interpretable in terms of the biomechanical processes that occur during running. This limits its practical use for injury prevention. To make it interpretable, a comparison of these features between the healthy and injured subjects must be made to establish the effect of the running biomechanics on these features. Second, the footwear in the experimental setup was not standardized, which limits the extent to which the influence of the footwear on the plantar pressure data can be determined. Third, all participants in this study had the same age (18–19 years old) and followed an identical minimal sports program, which might limit the applicability of this study to the wider public.

## 6 Conclusion and future work

The purpose of this study was, for both male and female, first-year students, (i) to predict lower extremity overuse injuries as accurately as possible and (ii) to identify the predictive loading features. We developed a model that can predict lower extremity overuse injuries for both men and women with an AUC of 0.639 and a Brier score of 0.201. Furthermore, we found that the higher pressures exerted on the forefoot are the most predictive for lower extremity overuse injuries and that foot areas from both the medial as the lateral side of the foot are needed. Additionally, we identified two kinds of predictive features: the angle of FFT coefficients and the coefficients of the autoregressive *AR* process. However, these features are not directly interpretable in terms of the biomechanical processes that occur during running, which makes it hard to interpret the predictions of the model. Future work that investigates the connection between these features and the biomechanical processes and compares them for the healthy and injured participants might make the model more interpretable.

## Data Availability

The raw data supporting the conclusion of this article will be made available by the authors, without undue reservation.

## Footnotes

1 The used code for calculating the 2D histogram-based mutual information can be found on https://gist.github.com/GaelVaroquaux/ead9898bd3c973c40429

2 For a complete list of the extracted features, we refer to https://tsfresh.readthedocs.io/en/latest/text/list_of_features.html

3 disclaimer: these definitions are based on the ones of the website of tsfresh: https://tsfresh.readthedocs.io/en/latest/text/list_of_features.html
